# Supplementary material for: Cross-Cultural Control–Value Mechanisms on the Detrimental Effect of Bullying on Mathematics Anxiety
Source: Behav Sci (Basel). 2025 Dec 19;16(1):3. doi: 10.3390/bs16010003 (PMC12837808; doi:10.3390/bs16010003)
Supplement: Supplementary file 1 [file behavsci-16-00003-s001.zip › behavsci-4030144-supplementary.pdf]

**SUPPLEMENTARY FILE**

## Main Variables

### Table S1

*Descriptive information for main variables*

| Main Variables | Item | Response Range |
|---|---|---|
| Bullying[1] | Student bullying/IDX | 1=Never or almost never<br>2=About monthly<br>3=About weekly |
| Mathematics Anxiety* | Mathematics makes me nervous | 1=Disagree a lot<br>2=Disagree a little<br>3= Agree a little<br>4= Agree a lot |
| Perceived Control for learning mathematics | Mathematics is more difficult for me than for many of my classmates<br>Mathematics is not one of my strengths<br>Mathematics is harder for me than any other subject | 1= Agree a lot<br>2= Agree a little<br>3= Disagree a little<br>4= Disagree a lot |
| Perceived Value for learning mathematics* | I learn many interesting things in mathematics<br>I look forward to mathematics class<br>I like to solve mathematics problems | 1=Disagree a lot<br>2=Disagree a little<br>3= Agree a little<br>4= Agree a lot |

*Note.* * Item(s) reverse coded for the variable. 1 Individual bullying items for this composite variable and their psychometric properties can be found in TIMSS & PIRLS International Study Center (2020)

## Covariates

### Table S2

*Descriptive information for covariates*

| Covariates | Item | Response Range |
|---|---|---|
| Gender | Students gender | 1=Girl; 2=Boy |
| Age | Student's age | Min. = 9 Max.= 19 |
| Parent education* | Parents' highest level of education | 1=Some primary, lower secondary or no school**<br>2= Lower secondary<br>3=Upper secondary<br>4= Post-secondary but not university<br>5= University or higher |
| Books at home | Number of books in your home | 1= 0-10 books**<br>2= 11-25 books<br>3= 26-100 books<br>4= 101-200 books<br>5= More than 200 books |
| Home possessions* | Do you have any of these things at your home? A computer or tablet | 0= No<br>1= Yes |
| | Do you have any of these things at your home? Study desk/table for your use | 0= No<br>1= Yes |
| | Do you have any of these things at your home? Your own room | 0= No<br>1= Yes |
| | Do you have any of these things at your home? Internet connection | 0= No<br>1= Yes |
| | Do you have any of these things at your home? Your own mobile phone | 0= No<br>1= Yes |
| Class size | Number of students in the class | Min. = 1 Max.= 117 |
| Disadvantaged School | Approximately what percentage of students in your school have the following backgrounds? Come from economically disadvantaged homes | 1= 0 to 10%**<br>2= 11 to 25%<br>3= 26 to 50%<br>4= More than 50% |
| Geographic area of school location | Immediate area of school location | 1=Urban<br>2=Suburban<br>3=City<br>4=Village<br>5=Rural** |
| School Safety * | I feel safe when I am at school | 1=Agree a lot<br>2=Agree a little<br>3=Disagree a little<br>4= Disagree a lot |
| Language spoken at home | How often do you speak <language of test> at home? | 1=Always<br>2=Almost always<br>3=Sometimes<br>4=Never |
| Parental expectations | Parental expectations for student achievement | 1=Very high<br>2=High<br>3=Medium<br>4=Low<br>5=Very low |

*Note.* * Item(s) reverse coded and recoded as binary variable ranged from 1=Yes to 0=No
** Reference category for dummy variables

**Handling Missing Data and Removal of Outliers**

PSM analysis perform better for large samples (Rubin, 1997) like TIMSS 2019 dataset; however, this dataset has missing values on multitude of variables. Conducting PSM analyses on covariates with missing values can confound the treatment effect estimation (D'Agostino & Rubin, 2000). The variables socioeconomic status, classroom size and disadvantaged school generally exhibited the highest incidence of missing values across countries. The examination of the missing data patterns indicated that the data was missing at random. The reporting of student families' socioeconomic status presents several challenges, including the varying and relative definitions of high and low socioeconomic status, the absence of student socioeconomic data within the school, and concerns surrounding privacy. The 'classroom size' variable was likely reported by educators who may have opted to leave it unfilled if they were responsible for multiple classrooms. The other variables exhibited a minimal percentage of missing values. In conjunction with the previous argument pertaining the pattern of missing data and theoretical rationales, the missing data mechanism was considered ignorable, missing at random. The covariates with about one percent missing values were replaced by their mean values. Because the missing values of other variables consisted of continuous, ordinal and binary variables, multivariate imputation by chained equations (MICE) technique was utilized in Stata. This technique performs a series of univariate models that subsequently uses posterior predictive distribution of imputed data on a variable-by-variable bases and does not require a multivariate normal distribution (Bouhlila & Sellaouti, 2013). By performing the MICE technique, five imputed datasets were produced for each country dataset.

The variables encompassing gender, age, the number of books available at home, possession of a personal computer or tablet by the student, ownership of a personal desk, individual student room availability, internet accessibility, and mobile phone ownership exhibited minimal percentages of missing data. The absent values were substituted employing the mean replacement method. Furthermore, the missing values associated with the constructs of mathematics self-concept and student expectations were similarly addressed through mean replacement. These variables, alongside average mathematics achievement, which presented no missing values, were utilized as covariates to enhance the accuracy of the estimation of missing values for the variables of interest through the application of the MICE technique within Stata (See Tables S3-S9).

Finally, multivariate outliers can distort population parameter estimates (Leys et al., 2018). To detect multivariate outliers, minimum covariance determinant estimator (Verardi & Dehon, 2010), which is a more robust variant of the Mahalanobis distance, was performed separately on outcome variables and covariates in Stata software. To prevent ecological validity of the representative datasets, only outliers (i.e., robust distance >10) that show the characteristics of random responders were excluded from datasets. The characteristics of random responders that are applied to the decisions on outliers are being male, having fewer books at home and rarely or never speaking the test language (Chen et al., 2023).

**Table S3**

*Sample sizes of the imputed datasets after data cleaning*

| | Initial N | Imputed Dataset 1 | Imputed Dataset 2 | Imputed Dataset 3 | Imputed Dataset 4 | Imputed Dataset 5 |
|---|---|---|---|---|---|---|
| Chile | 4115 | 4106 | 4107 | 4107 | 4107 | 4113 |
| Singapore | 4853 | 4844 | 4842 | 4843 | 4842 | 4853 |
| South Africa | 20829 | 20598 | 20827 | 20829 | 20237 | 20696 |

| | | | | | | |
|---|---|---|---|---|---|---|
| Sweden | 3996 | 3980 | 3977 | 3979 | 3982 | 3972 |
| Turkey | 4077 | 4077 | 4031 | 4031 | 4031 | 3982 |
| U.S.A. | 8698 | 8641 | 8642 | 8641 | 8692 | 8688 |

**Table S4**

*Before and after MICE and removal of outliers: Descriptive statistics and missing data for Chile*

| | Before | | After |
|---|---|---|---|
| | % Missing | Avg. | Pooled Avg. |
| manx | 2.84 | 2.327 | 2.330 |
| pc1 | 2.16 | 2.650 | 2.647 |
| pc2 | 2.84 | 2.388 | 2.386 |
| pc3 | 2.04 | 2.629 | 2.626 |
| pv1 | 2.07 | 2.961 | 2.963 |
| pv2 | 1.99 | 2.515 | 2.515 |
| pv3 | 1.82 | 2.117 | 2.115 |
| pareduc | 14.65 | 3.737 | 3.712 |
| sch_disadv | 8.46 | 3.120 | 3.116 |
| cl_size | 40.92 | 34.137 | 33.876 |

**Table S5**

*Before and after MICE and removal of outliers: Descriptive statistics and missing data for Singapore*

| | Before | | After |
|---|---|---|---|
| | % Missing | Avg. | Pooled Avg. |
| manx | 0.16 | 2.629 | 2.629 |
| pc1 | 0.14 | 2.608 | 2.608 |
| pc2 | 0.21 | 2.465 | 2.467 |
| pc3 | 0.10 | 2.732 | 2.733 |
| pv1 | 0.12 | 3.036 | 3.037 |
| pv2 | 0.08 | 2.754 | 2.755 |
| pv3 | 0.08 | 2.631 | 2.632 |
| pareduc | 30.83 | 4.168 | 4.114 |
| sch_disadv | 0.00 | 1.870 | 1.869 |
| cl_size | 3.63 | 33.816 | 33.878 |

**Table S6**

*Before and after MICE and removal of outliers: Descriptive statistics and missing data for South Africa*

| | Before | | After |
|---|---|---|---|
| | % Missing | Avg. | Pooled Avg. |
| manx | 3.01 | 2.724 | 2.728 |
| pc1 | 2.32 | 2.339 | 2.334 |
| pc2 | 3.58 | 2.346 | 2.342 |
| pc3 | 2.56 | 2.312 | 2.306 |
| pv1 | 2.79 | 3.328 | 3.327 |
| pv2 | 2.05 | 3.027 | 3.026 |
| pv3 | 2.05 | 3.111 | 3.111 |
| pareduc | 20.53 | 3.495 | 3.494 |
| sch_disadv | 5.29 | 3.553 | 3.563 |
| cl_size | 14.20 | 47.582 | 47.669 |

**Table S7**

*Before and after MICE and removal of outliers: Descriptive statistics and missing data for South Africa*

| | **Before** | | **After** |
|---|---|---|---|
| | **% Missing** | **Avg.** | **Pooled Avg.** |
| manx | 4.35 | 2.084 | 2.088 |
| pc1 | 3.83 | 2.761 | 2.749 |
| pc2 | 4.05 | 2.423 | 2.419 |
| pc3 | 4.08 | 2.932 | 2.921 |
| pv1 | 3.43 | 2.595 | 2.595 |
| pv2 | 3.10 | 2.430 | 2.430 |
| pv3 | 3.25 | 2.166 | 2.175 |
| pareduc | 43.49 | 4.318 | 4.216 |
| sch_disadv | 7.46 | 1.826 | 1.825 |
| cl_size | 13.59 | 24.732 | 24.707 |

**Table S*8***

*Before and after MICE and removal of outliers: Descriptive statistics and missing data for Turkey*

| | Before | | After |
|---|---|---|---|
| | % Missing | Avg. | Pooled Avg. |
| manx | 2.85 | 2.573 | 2.577 |
| pc1 | 1.96 | 2.649 | 2.649 |
| pc2 | 2.45 | 2.642 | 2.641 |
| pc3 | 2.06 | 2.312 | 2.307 |
| pv1 | 3.14 | 3.195 | 3.195 |
| pv2 | 2.82 | 2.953 | 2.949 |
| pv3 | 2.72 | 2.545 | 2.541 |
| pareduc | 7.85 | 2.658 | 2.668 |
| sch_disadv | 0.52 | 3.081 | 3.077 |
| cl_size | 0.52 | 29.571 | 29.573 |

**Table S9**

*Before and after MICE and removal of outliers: Descriptive statistics and missing data for the U.S.*

| | Before | | After |
|---|---|---|---|
| | % Missing | Avg. | Pooled Avg. |
| manx | 7.21 | 2.231 | 2.238 |
| pc1 | 6.94 | 2.738 | 2.728 |
| pc2 | 7.71 | 2.566 | 2.555 |
| pc3 | 7.12 | 2.758 | 2.747 |
| pv1 | 6.30 | 2.848 | 2.852 |
| pv2 | 5.89 | 2.554 | 2.554 |
| pv3 | 5.93 | 2.468 | 2.471 |
| pareduc | 27.77 | 4.095 | 4.041 |
| sch_disadv | 11.23 | 3.072 | 3.086 |
| cl_size | 15.79 | 31.768 | 31.752 |

**Creating Composite Latent Variables: Perceived control and perceived value**

Initially, the internal consistency of the instruments measuring perceived control and perceived value (specifically in the context of mathematics) was assessed (refer to Table S10). The findings regarding reliability exhibited a range from acceptable to high levels.

**Table S10**

*Cronbach's alpha reliability coefficients*

| | Perceived Control Items | Perceived Value Items |
|---|---|---|
| Chile | 0.758 | 0.825 |
| Singapore | 0.865 | 0.816 |
| South Africa | 0.710 | 0.762 |
| Sweden | 0.8851 | 0.840 |
| Turkiye | 0.790 | 0.768 |
| United States | 0.860 | 0.845 |

Confirmatory factor analysis (CFA) was conducted on Mplus statistical software using individual imputed datasets. Student-level weight variable was defined in the model. Additionally, since students were nested in the schools, school ID variable was used as a cluster variable that required the use of 'TYPE = COMPLEX ' analysis mode. Robust maximum likelihood (MLR) estimator was used to obtain estimates that are robust to non-normality. Using Hu and Bentler's (1999) strict criteria for model fit (i.e., RMSEA<0.06; CFI and TLI >0.95; and SRMR<0.08), CFA analyses of all imputed datasets showed very good fit to the data without specification of any error correlation. All standardized factor loadings were satisfactory (See Table S11). The results provided reliability and validity evidence that the scale has a univariate structure.

**Table S11**

*Average standardized factor loadings*

| Country | Factor Loadings | | | | | |
|---|---|---|---|---|---|---|
| | PC1 | PC2 | PC3 | PV1 | PV2 | PV3 |
| Chile | 0.643 | 0.759 | 0.715 | 0.647 | 0.900 | 0.787 |
| Singapore | 0.768 | 0.903 | 0.810 | 0.668 | 0.872 | 0.760 |
| South Africa | 0.600 | 0.694 | 0.648 | 0.608 | 0.764 | 0.748 |
| Sweden | 0.776 | 0.873 | 0.781 | 0.738 | 0.880 | 0.789 |
| Turkey | 0.669 | 0.860 | 0.713 | 0.551 | 0.859 | 0.767 |
| U.S. | 0.779 | 0.856 | 0.822 | 0.678 | 0.893 | 0.828 |

**Propensity Score Matching**

Our treatment variable is the hypothetical assignment to being bullied ($W_i$) and the respective outcome variables are student mathematics anxiety ($M_i$), control appraisals ($C_i$) and value appraisals ($V_i$). In specifications of unconfoundedness of covariates, a pre-specified set of fixed-effect covariates ($X_i$) that can potentially impact the relationship between the treatment and outcome variables were selected and their imbalance was checked by formal tests including nonparametric Wilcoxon Rank Sum test (Mann-Whitney U test), standardized differences and visual inspection of their distribution before and after matching. An extensive number of theoretically or empirically relevant covariates, that were available in the TIMSS 2019 dataset, were included in the analyses to reduce bias and confounding.

Conditional on these $X_i$s, propensity scores (PS) for each participant is calculated using the equation

$$PS = Pr(W_i = 1|X_i)$$

where $W_i$ is the assignment into the treatment condition (i.e., either or not being bullied). Because the probability of getting into treatment is unknown in non-experimental designs (Rubin, 2001), these propensity scores are used to determine the probability of being assigned to

treatment group conditional on observed covariates $X_i s$. These propensity scores are calculated for each participants $i$ and $j$, and statistically balanced samples on specified covariates are selected into the treatment and control groups (i.e., $PS(x_i) \cong PS(x_j)$). As widely acknowledged among the scholars, when the sample size is sufficiently large, randomized-controlled trials eliminate selection bias and reveal balanced samples between treatment and control groups. The matching process, similarly, helps to estimate causal evidence by significantly eliminating sample selection bias (Guo & Frazer, 2015). Additionally, when a data obtained from complex sampling design that include sampling weights, as is the case for the TIMSS survey, it becomes imperative to integrate these weights into the PS analysis to mitigate bias arising from disparities in selection and/or response rates, which facilitates the estimation of a treatment effect that can be generalized to the broader population (Ridgeway et al., 2015). Therefore, total student weight was integrated into the calculation of propensity scores. Yet, because the degree of reduction in selection bias depends on the covariates observed, causal inferences cannot be made with the same certainty as with randomized allocation (Winship & Morgan, 2014) due to omitted variables. For this reason, to make more valid interpretations regarding the causal effect estimates obtained from the analyses, ex post sensitivity analyses, using sensatt command in Stata, are conducted to estimate the potential effect of unobserved covariates on the outcomes. Several assumptions of propensity score matching method must hold before conducting propensity score analyses on balanced samples and estimating causal effects: i) strong ignorability of treatment assignment condition (i.e., after accounting for the effects of covariates on the treatment and control groups, only the treatment itself explains the difference in outcomes between the groups) and ii) stable unit treatment value assumption (SUTVA; i.e., being in the treatment and control group does not influence individuals' response to the treatment), which can

be assessed by checking covariate balance between the treatment and control group, observing common support region of propensity scores of the groups and performing sensitivity analyses (Powell et al., 2019; Rosenbaum & Rubin, 1983). To improve balancing between treatment and control groups, the specification of interaction and higher-order terms in the propensity score model was performed, which is a common practice. The balance was assessed through i) percentage of bias calculations on unmatched and matched samples aiming for mean bias below 5% post-matching for effective covariate balancing, ii) t-tests for values of covariates before and after matching, aiming for non-significance and iii) sufficient overlap of propensity score distributions among treated and control groups.

Because this study focused on the average effect that occurs in mathematics anxiety for those who actually experienced bullying, average treatment effect on the treated (ATT) was calculated which can be formulated for the treated group ($W_i = 1$) as

$$ATT_{(bullied,manx)} = E(M_{1i}|W_i = 1) - E(M_{0i}|W_i = 1)$$

where $ATT_{(bullied,manx)}$ is ATT of being bullied on mathematics anxiety, and M refer to the expected mathematics anxiety for treated (1) and untreated (0) groups. In other words, ATT value captures the average difference between the outcome (mathematics anxiety levels) of bullied students and the counterfactual outcome that would have occurred had these identical individuals not experienced bullying, after conditioning observed covariates used in the matching. The same formulation applies to the examination of control and value appraisals.

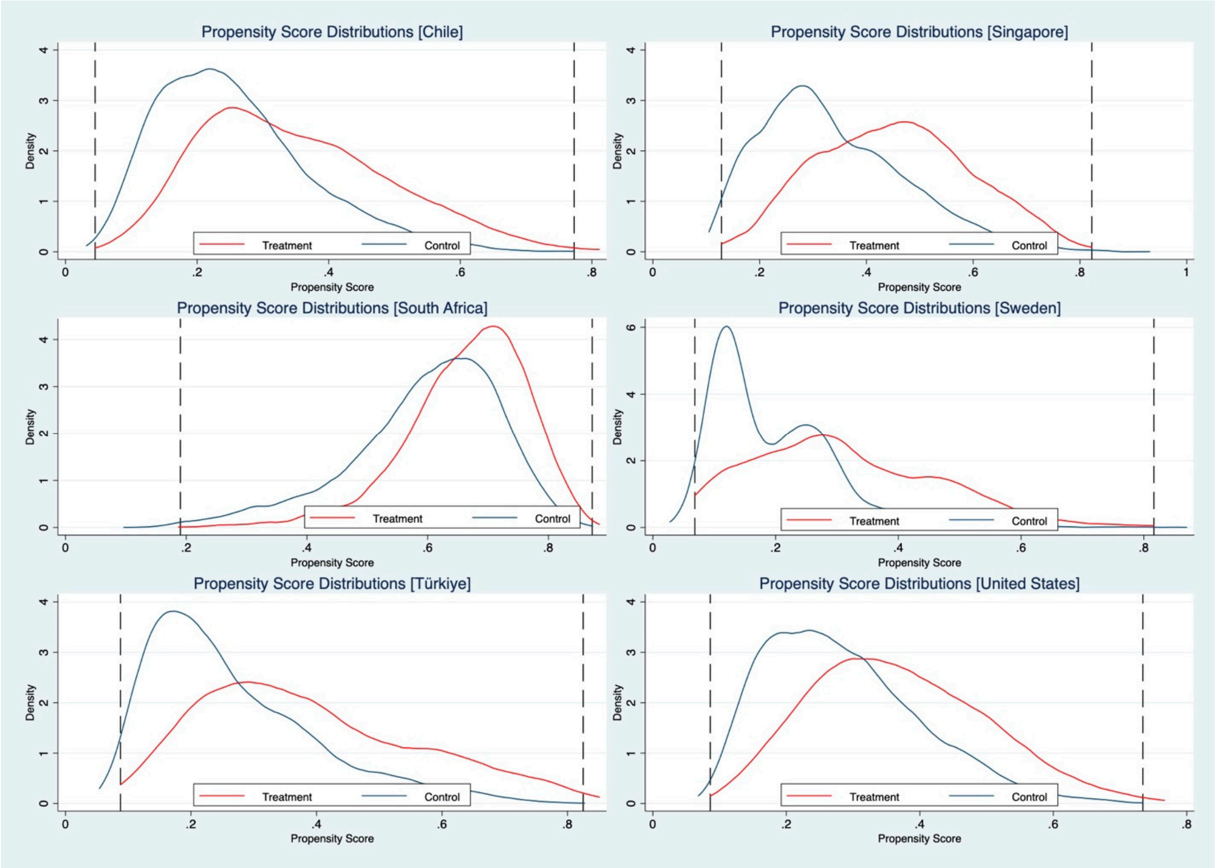


**Figure S1.** Propensity score distributions by countries.

## Country-Specific Modifications for Balancing Covariates

| | |
|---|---|
| Chile | - Age, feelsafeschool, math variables were squared<br>-Interaction terms added: sch_disadv4*class_size, parented_uni*sch_disadv4 |
| Singapore | -Home location variable only includes urban dwellers. Other categories were excluded.<br>-Only the class size variable was squared<br>-Interaction terms added: sch_disadv4*cl_size, ownroom*sch_disadv3, sch_disadv3*cl_size, parented_uni*sch_disadv4 |
| South Africa | -The same modification as Chile |
| Sweden | -The same modification as Chile, except feelsafesch and diff_lang_home were squared |

| | |
|---|---|
| Turkey | -The same modification as Chile |
| U.S. | -The same modification as Chile |

**Robustness Check**

To assess the robustness of the treatment effect of bullying on the outcomes, the results of kernel matching were compared with nearest neighbor matching with a caliper of 0.1 and caliper matching with a caliper size of 0.1 were calculated (See Table S12).

**Table S12**

*Treatment effect estimation using different matching techniques*

| | | Chile | Singapore | South Africa | Sweden | Türkiye | USA |
|---|---|---|---|---|---|---|---|
| Kernel matching | Manx | 0.075 | 0.077 | 0.058 | 0.125 | 0.101 | 0.091 |
| | | (0.017) | (0.014) | (0.007) | (0.020) | (0.019) | (0.012) |
| | PC | -0.023 | -0.040 | -0.030 | -0.048 | -0.108 | -0.061 |
| | | (0.014) | (0.014) | (0.006) | (0.017) | (0.016) | (0.011) |
| | PV | 0.008 | -0.005 | 0.001 | -0.020 | -0.029 | -0.020 |
| | | (0.014) | (0.011) | (0.005) | (0.016) | (0.014) | (0.010) |
| Nearest Neighbor matching | Manx | 0.074 | 0.069 | 0.061 | 0.119 | 0.103 | 0.091 |
| | | (0.017) | (0.014) | (0.008) | (0.020) | (0.018) | (0.012) |
| | PC | -0.025 | -0.032 | -0.016 | -0.037 | -0.099 | -0.056 |
| | | (0.013) | (0.013) | (0.006) | (0.017) | (0.016) | (0.011) |
| | PV | 0.006 | -0.003 | 0.008 | -0.021 | -0.028 | -0.019 |
| | | (0.013) | (0.011) | (0.005) | (0.016) | (0.014) | (0.010) |
| Caliper matching | Manx | 0.072 | 0.078 | 0.058 | 0.129 | 0.098 | 0.072 |
| | | (0.023) | (0.019) | (0.010) | (0.028) | (0.026) | (0.017) |
| | PC | -0.017 | -0.034 | -0.029 | -0.041 | -0.097 | -0.058 |
| | | (0.018) | (0.018) | (0.008) | (0.024) | (0.022) | (0.015) |
| | PV | 0.007 | -0.009 | 0.001 | -0.015 | -0.032 | -0.021 |
| | | (0.018) | (0.014) | (0.006) | (0.022) | (0.019) | (0.013) |

Manx: mathematics anxiety; PC: perceived control; PV: perceived value. Standard errors are given in parentheses.
